# PPARγ: a transfactor for Th-cell subsets

**DOI:** 10.3389/fimmu.2026.1937624

**Published:** 2026-10-23

**Authors:** Sihang Chen, Ming Chu

**Affiliations:** 1 Department of Immunology, School of Basic Medical Sciences, Peking University Health Science Center, Beijing, China; 2 Division of Life Sciences, Faculty of Science, The Chinese University of Hong Kong, Hong Kong, Hong Kong SAR, China

**Keywords:** PPARγ, Tfh, Th1, Th17, Th2, Th9, Treg

## Abstract

Peroxisome proliferator-activated receptor γ (PPARγ) is a ligand-activated nuclear transcription factor that governs helper T cell (Th) immunometabolism and functional differentiation by regulating gene transcription, cellular metabolism, and cytokine microenvironment. In Th1 cells, PPARγ primarily regulates interferon-γ (IFN-γ) secretion. Within Th2 cells, it influences GATA-binding protein 3 (GATA3) expression and provides metabolic support for normal cellular functions. In Th17 cells, it inhibits RAR-related orphan receptor γt (*RORγt*) transcription and suppresses inflammatory signals, thereby alleviating Th17-mediated inflammatory responses. In Tregs, PPARγ aids in cell differentiation by activating forkhead box P3 (*Foxp3*) transcription and inducing metabolic reprogramming. In Th9 cells, PPARγ promotes the secretion of IL-9. Within Tfh cells, the deficiency of PPARγ leads to an increase in B cell leukemia/lymphoma 6 (*Bcl-6*) expression. Notably, PPARγ agonists and antagonists effectively preserve Th-cell homeostasis by targeting PPARγ-mediated metabolic and signaling pathways. This review summarizes PPARγ as a crucial transcription factor governing Th-cell responses and elucidates its underlying molecular mechanisms. Based on the evidence predominantly derived from animal models, it lays a foundation for translational research of PPARγ-targeted immunotherapy.

## 1 Introduction

PPARγ is a member of the peroxisome proliferator-activated receptor superfamily. Compared to other PPAR subtypes, PPARγ has been more extensively studied in mouse and human T cells (1). As a ligand-activated transcription factor, PPARγ is essential for transcriptional regulation. Mechanistically, upon activation by its cognate ligand, PPARγ heterodimerizes with retinoid X receptor α (RXR-α). The resulting PPARγ/RXR-α complex binds to peroxisome proliferator-responsive elements (PPREs) located in the promoter regions of its target transcripts. This binding event induces alterations of chromatin structure and facilitates the recruitment of the transcriptional apparatus, culminating in alterations in target transcriptional output (2). Through this specific transcriptional regulatory cascade, PPARγ exerts profound effects on Th-cell homeostasis and fundamental cellular function (3).

Emerging paradigms in immunology indicate that lymphocyte activation is closely coupled to metabolic reprogramming (4). In Th-cells in particular, metabolic reprogramming plays a pivotal role in their differentiation and functional maintenance (4), relying on core metabolic pathways including fatty acid β-oxidation (FAO), oxidative phosphorylation (OXPHOS) (5) and glycolysis (6). PPARγ regulates enzymes involved in FAO, such as Carnitine palmitoyltransferase 1 (CPT1) (7). And it activates AMP-activated protein kinase (AMPK) (8). Additionally, PPARγ interacts with peroxisome proliferator-activated receptor gamma coactivator-1α (PGC-1α), enhancing the transcriptional control of genes related to mitochondrial biosynthesis and oxidative metabolism (9). Moreover, PPARγ modulates key transcription factors, including hypoxia-inducible factor-1α (HIF-1α), thereby influencing glycolytic flux in various cellular contexts to regulate cellular functions (6). These findings highlight essential functions of PPARγ in orchestrating Th-cell metabolic reprogramming and maintaining functional homeostasis, thereby establishing a robust foundation for investigating its therapeutic potential in immune-related disorders. The regulatory function of PPARγ in Th-cells, particularly concerning the differentiation and functional modulation of Th-cell subsets, has attracted increasing attention (10). Herein, we delve into the crucial implications of PPARγ in the lineage commitment and activities of Th-cell subsets, encompassing Th1, Th2, Th17, Tregs, Th9 and Tfh, with the aim of providing novel insights into the regulatory mechanisms of PPARγ during the activation of T cells and steering future investigations in this domain (**Tables 1**, **2**).

**Table 1:** The effects of PPARγ on different Th-cell subpopulations.

Th-cell subsets	Key targets of PPARγ	The regulatory effects of PPARγ	Effector cytokines	Ref
Th1	↓*STAT3* ↑*IκBα* ↑*Foxo1* ↑*Sirt1*	Inhibit Th1 differentiation and attenuate Th1 function	↓IFN-γ ↓TNF-α	(11–13)
Th2	↓*GATA3* ↑*ST2* ↑*CRTH2*	Inhibit Th2 differentiation but stabilize Th2 surface molecule	↓IL-4 ↓IL-5 ↓IL-13	(14–19)
Th17	↓*RORγt* ↓*HIF-1α* ↓*Sp1* ↓ROS ↓NF-κB	Inhibit Th17 differentiation and attenuate Th17 function	↓IL-17A ↓IL-17F ↓IL-22 ↓TNF-α	(20–31)
Treg	↑*Foxp3* ↑*CD36/CPT1* ↓*HIF-1α*	Promote Treg differentiation and stabilize Treg function	↑TGF-β ↑IL-10	(10, 28, 32–37)
Th9	↑mTORC1 ↑*SLC7A8*	Promote Th9 differentiation	↑IL-9	(38–40)
Tfh	↓*Bcl-6* ↑*IκBα* ↑*Foxo1* ↑*Sirt1*	Inhibit Tfh differentiation Sex-dependent	↓IL-21	(18, 41–43)

This table summarizes the metabolic-dependency patterns, key molecular targets, and functional outcomes of PPARγ in major CD4^+^ Th-cell subsets. PPARγ inhibits the differentiation of Th1, Th2 and Th17 effector cells, whereas it promotes differentiation and stabilizes function of Treg cells, accompanied by corresponding changes in signature cytokine production.

**Table 2:** PPARγ-mediated regulation of Th-cell subsets in mouse models.

Th-cell Subsets	Animal models	The regulatory effects of PPARγ	Ref
Th1	T-bet genetically modified mice	Elucidate T-bet-mediated Th1 lineage commitment	(44–47)
	Experimental autoimmune myocarditis (EAM) mouse model	Verify that PIO regulates Th1/Th2 balance and ameliorates autoimmune myocarditis	
	Experimental autoimmune encephalomyelitis (EAE) mouse model	PPARγ antagonist exacerbates neuroantigen-specific Th1 responses and aggravates inflammation in EAE	
Th2	Ovalbumin (OVA)-induced allergic airway inflammation mouse model	Verification of the inhibitory effect of PPARα/γ agonists on allergic inflammation	(48)
	Helminth infection-induced allergic airway inflammation mouse model	Demonstrate that PPARγ promotes type 2 immune responses in allergy and helminth infection	
Th17	Collagen-induced arthritis (CIA) mouse model	Elucidate leptin-PPARγ-mediated Th17 differentiation in obesity-associated rheumatoid arthritis Morin, a PPARγ agonist, blocks Th17 differentiation via inhibiting FASN	(22, 23, 27, 28, 31)
	Psoriasis mouse model	Baicalin activates PPARγ and inhibits the Wnt and Th17/IL-17 axes to treat psoriasis.	
	DSS-induced ulcerative colitis mouse model	Bufalin regulates the intestinal microbiota and the Th17/Treg balance, and inhibits the MAPK/NF-κB pathway. Stigmasterol restores the Treg/Th17 balance through the butyric acid-PPARγ axis	
	Crohn’s-like colitis mouse model	Bergapten regulates mitochondrial autophagy and Th17/Treg balance through PPARγ/NFκB	
	High-fat diet mouse model	Immunometabolism research in adipose tissue	
Treg	DSS-induced ulcerative colitis mouse model	Stachyose activates the butyrate-PPARγ pathway and remodels Treg/Th17 balance to alleviate colitis	(33, 34, 49–52)
	HDM-induced allergic asthma mouse model	mTOR/PPARγ axis alleviates allergic asthma	
	OVA-induced allergic rhinitis mouse model	PGE2 inhibits Treg differentiation through PPARγ PIO upregulates the proportion of CD4^+^Foxp3^+^ Tregs in a dose-dependent manner	
	Ulcerative colitis rat model	Sishen Pill regulates gut microbiota and restores Treg/Th17 balance for UC therapy	
	Tumor-bearing mouse mode	Tumor-derived ammonia metabolism regulates mitochondrial function in Tregs	
	High-fat diet mouse model	PPARγ drives the enrichment and phenotypic maintenance of adipose tissue Tregs Co-transfection of PPARγ and Foxp3 validates lipid metabolic genes	
Th9	N/A	N/A	N/A
Tfh	PPARγ knockout mouse model	PPARγ deficiency upregulates Bcl-6 expression	(41)

This table summarizes *in vivo* evidence of PPARγ regulating helper T cells from genetically modified and disease mouse models.

## 2 Molecular basis of PPARγ: structure, ligands and signaling mechanisms

### 2.1 Structural domains of PPARγ

The human *PPARγ* gene is located on chromosome 3p25 and generates multiple splice variants, the most functionally important being *PPARγ_1_* and *PPARγ_2_* (53). PPARγ_1_ ubiquitously expresses in hepatic tissue, and PPARγ_2_ specifically enriches in mature adipocytes with an extra 30 amino acid extension at the N-terminus (54).

Sharing a conserved structural layout with other PPAR isoforms, PPARγ contains four key functional regions (55). The N-terminal A/B domain carries activation function-1 (AF-1) and enables ligand-independent transcriptional activation. The A/B domain differs between PPARγ_1_ and PPARγ_2_; the extra 28 residues in *PPARγ_2_* enhance its transactivation potential in preadipocytes (54). The C domain is defined as DNA-binding domain (DBD) for DNA interaction. The DBD is the most conserved region among nuclear receptors and comprises two zinc-finger motifs, each coordinating a Zn^2+^ ion. The first zinc finger recognizes the consensus half-site AGGTCA within PPREs, while the second mediates dimerization, specifically heterodimerization with the RXR (56). The D domain works as a hinge segment connecting adjacent domains; the E/F domain accommodates Activation Function-2 (AF-2) and mediates ligand-dependent activation responses (57). The C-terminal ligand-binding domain (LBD) is the largest and most functionally complex region. The PPARγ LBD adopts a canonical nuclear receptor fold and is arranged in a three-layer antiparallel helical sandwich surrounding a four-stranded β-sheet. Upon agonist binding, H12 repositions to create a coactivator-binding surface (58).

The transcriptional activity of *PPARγ* is determined by the conformational change of the AF-2 functional helix H12 within the LBD (59). Previous structural studies have focused on the classic orthosteric ligand pocket. Recent high-resolution crystallography revealed two key novel structural findings: an independent secondary allosteric pocket non-competitive with thiazolidinediones (TZDs) (60), and co-crystal dual-ligand conformations of covalent antagonists plus small-molecule modulators (61). This binding mode stably anchors helix 12 to lock *PPARγ* in a transcriptionally repressive state and promotes assembly of the PPARγ Nuclear receptor corepressor 1 (NCoR1) repressive ternary complex (62). The different binding patterns may result in distinct conformations, which ultimately reshape the transcriptional regulatory network within T cells, thereby regulating the differentiation and functional balance of various Th-cell subsets such as Tregs, Th1, Th2, Th17 cells and so on.

### 2.2 Endogenous and synthetic ligands

PPARγ activity is tightly controlled by two major categories of ligands: endogenous lipid metabolites and artificially designed synthetic small molecules (63). Endogenous ligands mainly consist of polyunsaturated fatty acids (Docosahexaenoic acid and linoleic acid), oxidized phospholipids, nitroalkanes and cyclopentenone prostaglandins, among which 15-deoxy-Δ^12^,^14^-prostaglandin J_2_ (15d-PGJ_2_) is the most potent physiological agonist (64, 65).

Synthetic PPARγ modulators are broadly categorized into agonists and antagonists. TZDs, such as pioglitazone and rosiglitazone, serve as full orthosteric agonists. These compounds stabilize the active spatial conformation of the LBD alongside the AF-2 surface that interacts with coregulatory proteins, thereby stimulating PPARγ-dependent transcriptional programs (66). PPARγ partial agonists adopt unique ligand-binding conformations, trigger selective transcriptional programs of target genes, and exhibit fewer metabolic toxicities compared to full TZD agonists (67). GW9662 and T0070907 represent covalent PPARγ antagonists that react with Cysteine-285 within the orthosteric pocket via nucleophilic substitution, thereby disrupting the binding of prototype PPARγ agonist ligands (61).

### 2.3 Signaling mechanisms

As a nuclear receptor transcription factor, PPARγ executes canonical genomic transactivation (**Figure 1A**). Upon ligand binding, PPARγ undergoes a conformational rearrangement that facilitates heterodimerization with RXR-α and the displacement of co-repressor complexes, such as NCoR/SMRT (68, 69). Then, the PPARγ/RXR-α heterodimer binds to PPREs in target gene promoters and recruits co-activator complexes, leading to chromatin remodeling and transcriptional activation (2, 69). Abundant genomic and transcriptomic evidence has confirmed that PPARγ acts as a key transcriptional regulator governing adipogenic differentiation and metabolic homeostasis. More than one hundred functional target genes have been identified in mammalian cells and tissues (70). Functionally, this canonical trans-activation program primarily drives the expression of a panel of core downstream genes closely associated with Th-cellular metabolism and immune homeostasis. Representative targets include adipogenic markers such as *C/EBPα*, aP2 (*FABP4*), and adiponectin *(ADIPOQ*). These factors cooperatively orchestrate adipocyte differentiation, lipogenesis, and lipid droplet storage (67).

**Figure 1:**
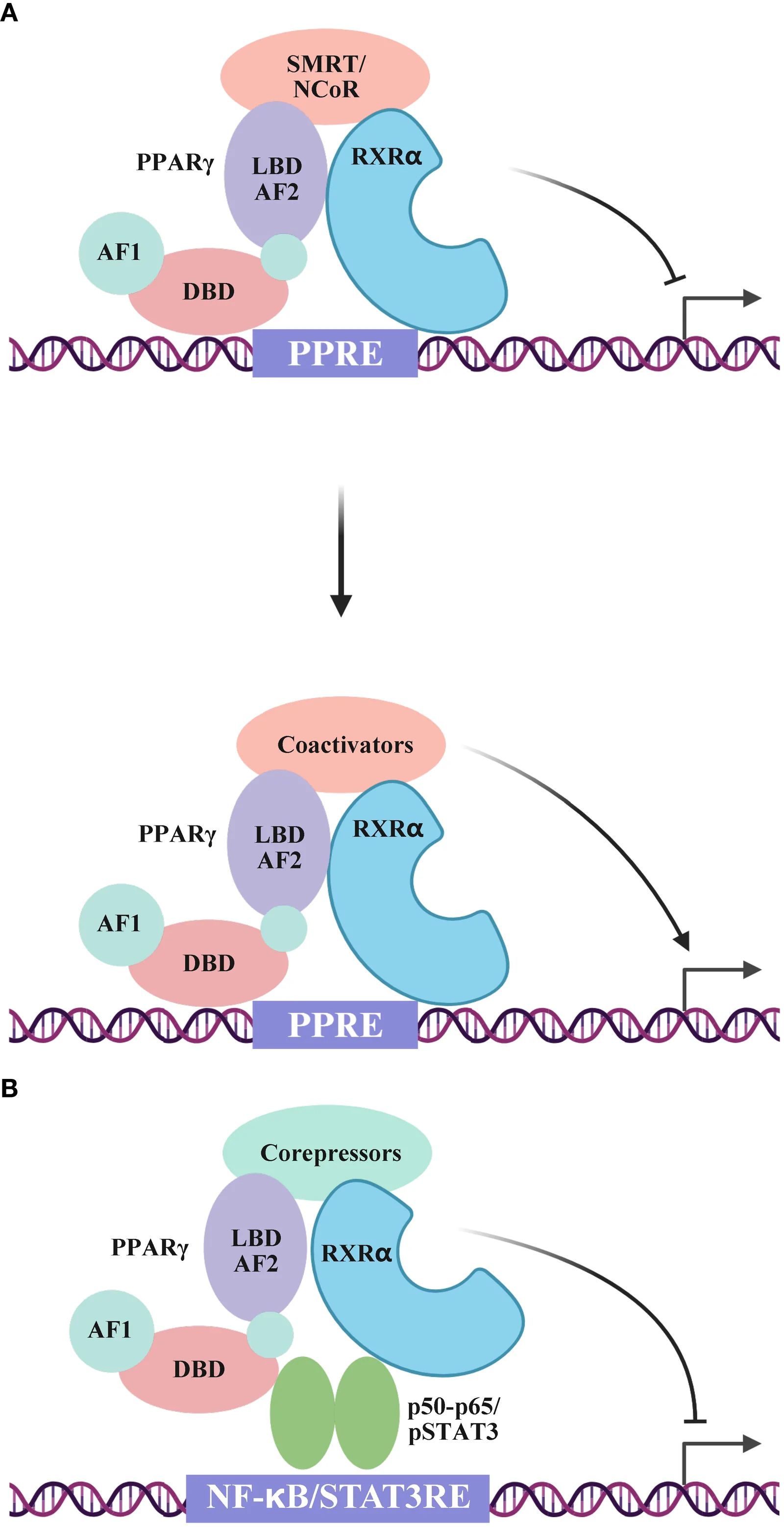
**(A)** Canonical signaling mechanisms of PPARγ. This schematic illustrates the transcriptional regulation mechanism of the PPARγ-RXRα heterodimer on target genes: PPARγ forms a heterodimer with RXRα, binding to the PPRE response element on DNA via the DBD domain of PPARγ. The protein contains an AF1 domain, a DBD, and an LBD ligand-binding domain harboring the AF2 motif. **(B)** Non-Canonical signaling mechanisms of PPARγ. This schematic diagram illustrates the cross-inhibition pattern of the PPARγ-RXRα heterodimer on the transcription mediated by NF-κB (p50-p65) and pSTAT3. The p50-p65 heterodimer represents the predominant functional complex of canonical NF-κB signaling. pSTAT3 denotes phosphorylated signal transducer and activator of transcription 3. Response element (RE) refers to conserved cis-acting DNA sequences recognized by transcription factors to modulate gene transcription.

PPARγ also exerts non-canonical transrepression effects (**Figure 1B**). Numerous mechanistic studies have demonstrated that this transcription-independent inhibitory mode constitutes the major mechanism underlying the broad functions of PPARγ in multiple immune cell types (63). For example, PPARγ can physically interact with nuclear factor-κB (NF-κB), signal transducer and activator of transcription 3 (STAT3), inhibiting their pro-inflammatory transcriptional activity independently of DNA binding (63, 71). This inhibitory cascade markedly reduces the expression of pro-inflammatory cytokines (TNF-α, IL-6, IL-1β), chemokines (72). Additionally, PPARγ can undergo post-translational modifications including phosphorylation, SUMOylation, and acetylation, which fine-tune its activity, stability, and subcellular localization in response to extracellular signals (73).

## 3 PPARγ in Th1 cells

The differentiation and development of Th1 cells are programmed processes regulated by the coordinated action of T-cell receptor (TCR) signaling, cytokine signals, and core transcription factors. Upon activation by TCR and co-stimulatory signals, naive CD4^+^ T cells are exposed to microenvironmental IFN-γ, which induces T-bet expression; T-bet in turn drives robust secretion of IFN-γ, forming a positive feedback loop that stabilizes the Th1 lineage (44), while simultaneously suppressing the transcriptional programs of other helper T cells, ultimately leading to high secretion of IFN-γ and acquisition of Th1 effector functions (11).

PPARγ exerts a modulatory function in the lineage commitment and functional homeostasis of Th1 cells (**Figure 2**). At the cell-intrinsic transcriptional level, PPARγ can decrease IFN-γ expression by interacting with *STAT3* to repress its transcriptional activity, as well as preserving the expression and stability of NF-κB-associated inhibitory molecules including inhibitor of nuclear factor-κB α (*IκBα*), silent information regulator 1 (*Sirt1*), and forkhead-box protein O1 (*Foxo1*). These combined actions consequently suppress the NF-κB pathway and attenuate the pro-inflammatory functions of Th1 cells (12, 41, 45). Beyond cell-autonomous transcriptional control, PPARγ shapes Th1 responses by remodeling extracellular signaling and immune microenvironment. PPARγ agonists diminish IL-12-initiated Janus kinase-STAT signaling and Cluster of differentiation 40 (CD40) pathways, resulting in reduced Th1 differentiation (46, 47, 74). In addition, PPARγ agonists can also inhibit the dose-dependent secretion of C-X-C motif chemokine ligand9 (CXCL9) and C-X-C motif chemokine ligand11 (CXCL11) induced by IFN-γ/TNF-α, thereby eliminating the chemotactic signals that facilitate Th1 cell recruitment (75, 76). Moreover, the activation of PPARγ receptors decreases the elaboration of Th1-derived inflammation-driving mediators, including TNF-α and IL-1, while simultaneously upregulating anti-inflammatory factors exemplified by IL-10 (13). PPARγ activation enhances adiponectin secretion, which directly inhibits Th1 cell responses and Th1 cell-dependent pro-inflammatory pathways, thereby indirectly alleviating Th1-mediated inflammation (77).

**Figure 2:**
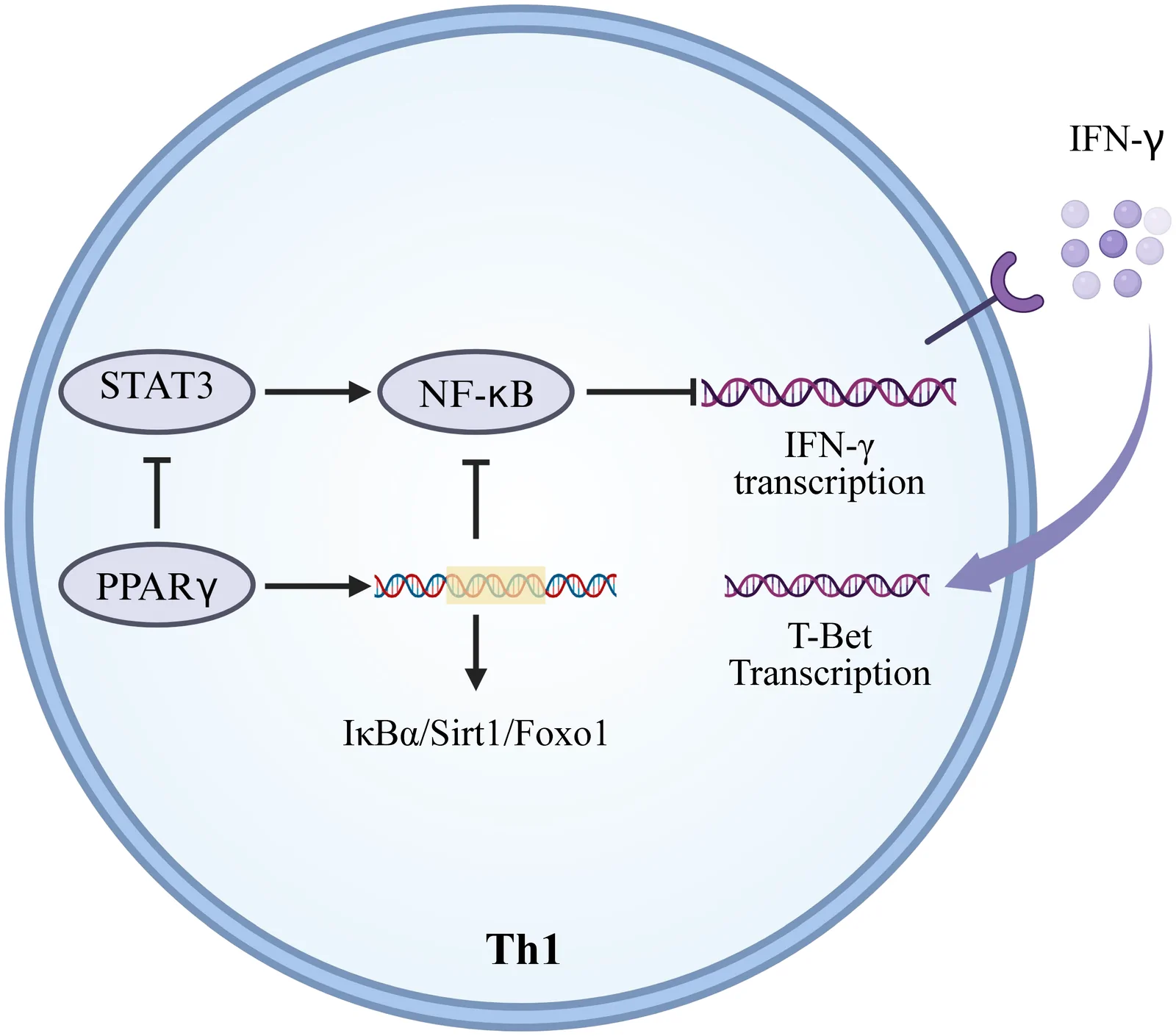
PPARγ in Th1 cells. This schematic diagram illustrates the transcriptional regulatory network mediated by PPARγ in Th1 cells: extracellular IFN-γ stimulation induces intracellular T-bet transcription, further promoting *IFN-γ* gene expression, establishing a positive feedback inflammatory loop in Th1 cells; STAT3 can enhance the activity of the NF-κB signal, driving *IFN-γ* transcription; PPARγ, on the one hand, directly inhibits *STAT3*, and on the other hand, maintains the expression of *IκBα*, *Sirt1*, and *Foxo1*, multi-levelly inhibiting NF-κB, ultimately limiting *IFN-γ* transcription and negatively regulating the pro-inflammatory function of Th1 cells.

Interestingly, PPARγ also serves as a modulator of Th1 cells under specific combined interventions. The pioglitazone (PIO) works synergistically with mesenchymal stem cell-derived exosomes to upregulate IFN-γ while downregulating IL-4 and IL-17. This interaction promotes Th1 cell activation, proliferation, and function by optimizing the cytokine milieu (78). Additionally, PPARγ enhances the expression of molecules associated with Th1 polarization by inhibiting M2 macrophage activation and alleviating the suppression of M1 macrophages (79).

## 4 PPARγ in Th2 cells

In Th2 cells, IL-4 activates STAT6, which facilitates the transcription of *GATA3* while sustaining IL-4 production (80). STAT6 serves as an upstream promoter of PPARγ, enhancing its transcriptional activity (81). Consequently, PPARγ is specifically and profoundly expressed in Th2 cells (82).

PPARγ serves a pivotal role in maintaining stable proliferation and supporting the function of Th2 cells **Figure 3**. Genome-wide CRISPR knockout library screening has validated that PPARγ functions as a core transcriptional regulator of canonical effector genes in Th2 cells, controlling the expression of GATA3 (14, 15). PPARγ agonists significantly inhibited *GATA3* expression, suppressed Th2 differentiation, and suppressed the expression and release of IL-4, IL-5, and IL-13 (16–18). Interestingly, the regulation of IL-4 is context-dependent, with PPARγ deficiency leading to unstable *IL-4* expression (41, 48, 82). Apart from that, the regulatory effect of PPARγ on Growth-stimulation-expressed gene 2 (ST2) expression is particularly pronounced in lung tissue Th2 cells (15). And PPARγ can activate the expression of Chemoattractant receptor-homologous molecule expressed on Th2 cells (CRTH2) through transcriptional regulation, with CRTH2 serving as a key molecule mediating Th2 cell functions (19).

**Figure 3:**
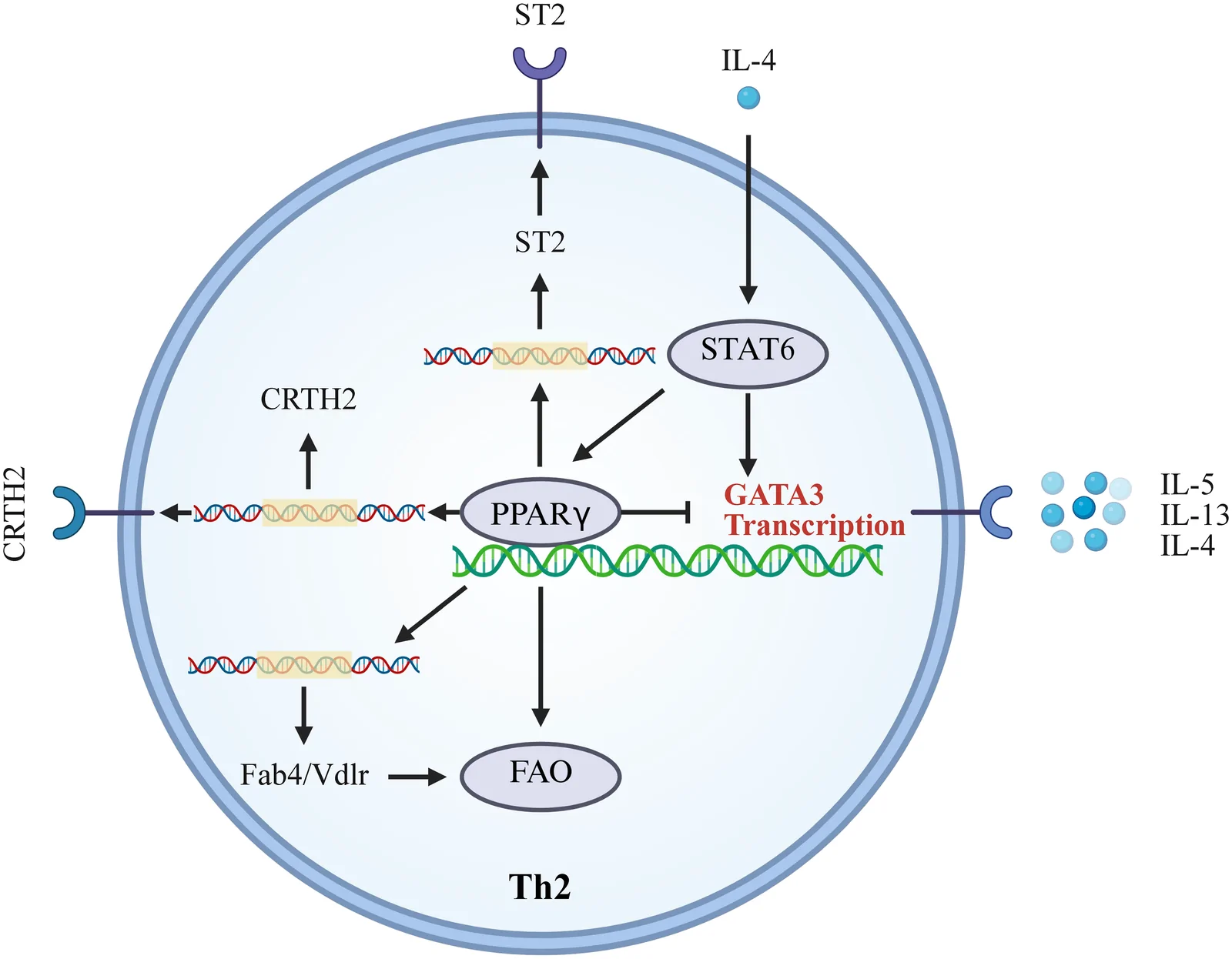
PPARγ in Th2 cells. This schematic diagram illustrates the immune-metabolic regulatory network mediated by PPARγ within Th2 cells. Extracellular IL-4 activates STAT6, inducing the expression of the lineage transcription factor GATA3, and driving the transcription of type 2 cytokines such as IL-4, IL-5, and IL-13; PPARγ, on the one hand, directly inhibits the transcriptional activity of *GATA3*, limiting the production of type 2 cytokines, and simultaneously upregulates expression of the ST2 and CRTH2; on the other hand, it transcribes and activates *Fabp4* and *Vldlr*, promoting FAO, and mediating the lipid metabolic reprogramming of Th2 cells.

Regarding lipid metabolism, mammalian target of rapamycin (mTOR)-mediated activation of PPARγ facilitates fatty acid uptake, which offers metabolic support for the late activation and tissue adaptation of Th2 cells (15, 83). PPARγ upregulates the transcript levels of lipid metabolism-associated genes, including fatty-acid-binding protein 4 (*Fabp4*) and very-low-density-lipoprotein receptor (*Vdlr*), in addition to genes vital to the effector functions of Th2 cells, e.g., *Il5* (15, 84).

## 5 PPARγ in Th17 cells

The conversion of naive T lymphocytes toward Th17 lineage is dependent on the nuclear transcription factor *RORγt* (85). Naive CD4^+^ T cells commit to the Th17 lineage under combined TGF-β and IL-6 stimulation via *STAT3*-dependent *RORγt* upregulation, producing signature cytokines including IL-17A, IL-17F and IL-22 to mediate host defense and autoimmune inflammation (86).

PPARγ acts as a suppressive transfactor of *RORγt*, inhibiting Th17 lineage commitment by repressing its transcription (**Figure 4**) (20–22). Under the PPARγ-mediated activation, the majority of CD4^+^ T cells do not express RORγt, resulting in a decreased Th17 cell population (20). Fu J and Pascual G et al. focused on transcriptional repression at the gene promoter level, revealing that PPARγ mediates recruitment of *NCoR/SMRT* complex onto the *RORγt* promoter, thereby inhibiting its transcription and suppressing IL-17A expression (87, 88). Leijten-vd G et al. investigated nuclear receptor ligand competition, showing that PPARγ competitively inhibits RORγt through shared ligands such as MRL-871 (89). De Oliveira et al. examined lipid metabolic regulation, demonstrating that PPARγ enhances FAO and represses lipid biosynthesis, which reduces the availability of lipid ligands essential for RORγt, thereby dampening its transcriptional level and inhibiting Th17 differentiation (90). Beyond canonical Th17-directed effects, this PPARγ-mediated repression of RORγt may also have indirect implications for related Th-cell subsets. Since Th22 cells rely on the cooperative action of AhR and RORC to initiate *IL-22* transcription the inhibitory effect of PPARγ on RORγt may partly influence IL-22 secretion by Th22 cells (82, 91).

**Figure 4:**
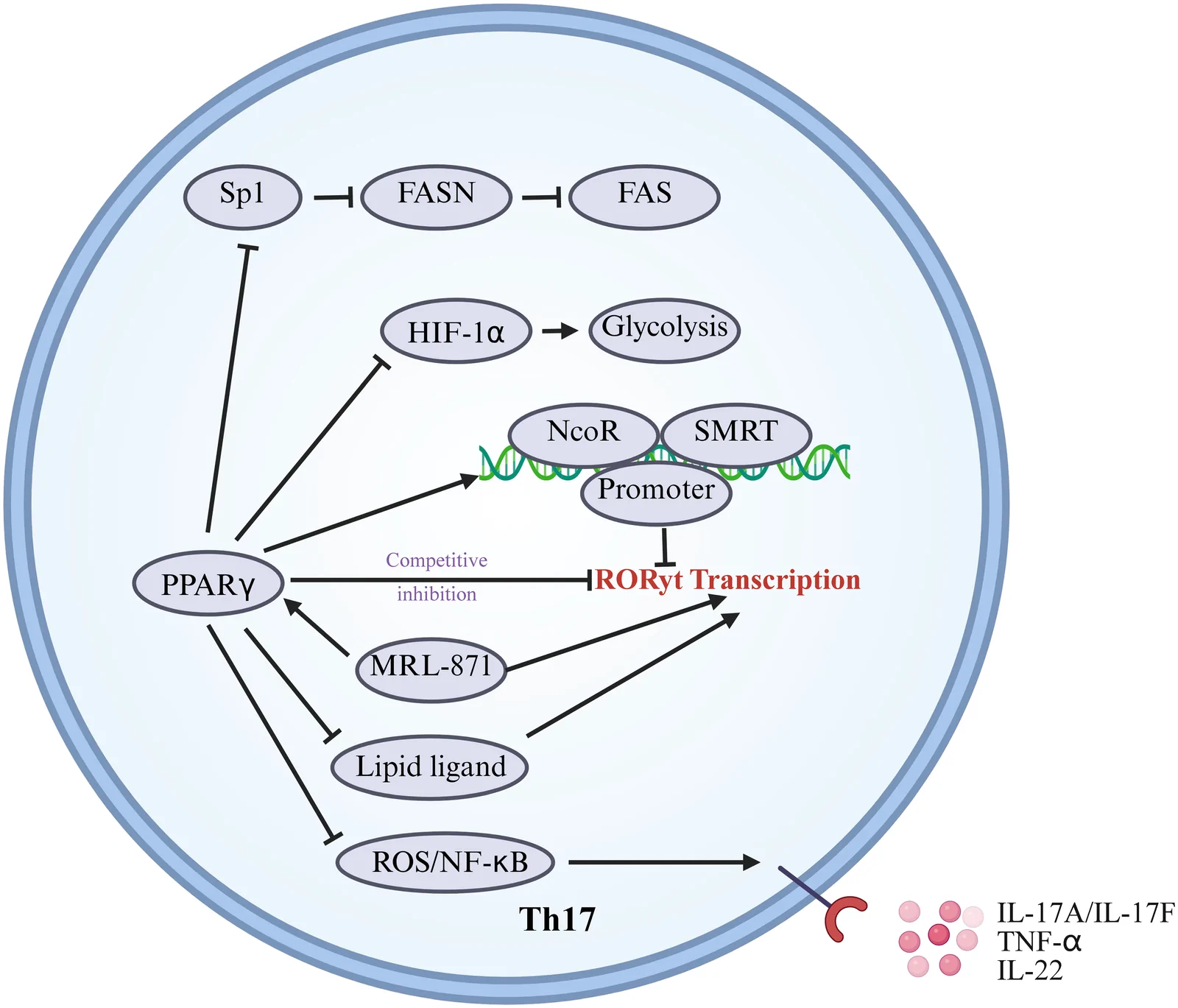
PPARγ in Th17 cells. This schematic diagram depicts the transcriptional and metabolic regulatory network mediated by PPARγ within Th17 cells. The key lineage transcription factor RORγt binds to the promoter of target genes, recruits the NcoR and SMRT complexes, and drives the transcription of Th17 characteristic pro-inflammatory factors such as IL-17A, IL-17F, TNF-α, and IL-22. PPARγ competitively inhibits the transcriptional activity of RORγt; the small molecule MRL-871 can directly inhibit RORγt and partially activate PPARγ, cooperatively blocking the Th17 effector program. In addition, PPARγ inhibits the ROS/NF-κB inflammatory pathway, inhibits glycolysis mediated by HIF-1α, and simultaneously inhibits Sp1, down-regulating the fatty acid *de novo* synthesis mediated by FASN-FAS, negatively regulating Th17 cells from multiple aspects including inflammation, glucose metabolism, and lipid metabolism.

In terms of the regulation of cytokines and the microenvironment, PPARγ can alleviate the inflammatory potency of Th17 subset via suppressing the production of key inflammatory cytokines (IL-17A, IL-17F and TNF-α) and Th17-induced keratinocyte IL-17C, which disrupts inflammatory signal transmission (23, 24). Kim et al. showed that PPAR-γ ligands, including ciglitazone, rosiglitazone, and troglitazone, suppressed Th17 lineage polarization coupled with IL-17/IL-22 production (25). PPARγ can promote polarization of macrophage to the M2-like anti-inflammatory and collaborates with the STAT6 pathway to suppress Th17 cell differentiation and pro-inflammatory functions, resulting in decreased secretion of pro-inflammatory effector cytokines alongside enhanced production of anti-inflammatory mediators exemplified by IL-10 (92). In terms of metabolic regulation, activated PPARγ in Th17 cells significantly suppresses Specificity protein 1 (Sp1) transcriptional activity through direct physical interaction, leading to reduced *FASN* mRNA levels and inhibited FAS. FAS is vital for the development of Th17 subset (26, 27). The survival of Th17 cells primarily depends on glycolysis (93), and PPARγ inhibits Th17 differentiation by decreasing glycolytic levels. Shuting W et al. demonstrated that the PPARγ antagonist GW9662 alleviates the suppression of Th17 cell differentiation by upregulating intracellular HIF-1α expression (28, 29). In addition to metabolic regulation, PPARγ mitigates the abnormal responses of Th17 cells through its interactions with inflammation-related signaling pathways (94). PPARγ can attenuate the inflammatory response of Th17 cells by suppressing reactive oxygen species (ROS) production. It inhibits NADPH oxidase while upregulating the expression of antioxidant proteins, thereby disrupting the vicious cycle between ROS-mediated inflammatory activation and exacerbated oxidative stress (30). PPARγ also inhibits the phosphorylation and activation of NF-κB, which reduces its pro-inflammatory effects on Th17 cells (31).

## 6 PPARγ in Tregs

Regulatory T cells (Tregs) are a suppressive CD4^+^ T-cell subset marked by the lineage-defining transcription factor Foxp3 (32). They comprise thymus-derived Tregs (tTregs/nTregs), induced by intermediate-strength self-reactive TCR signals plus IL-2 during thymic selection, and peripherally-induced Tregs (pTregs) (95), which differentiate from naive CD4^+^ T cells; cooperative TGF-β-Smad and IL-2-STAT5 signaling initiates *Foxp3* transcription to establish the Treg lineage (96).

Following heterodimerization with RXR-α, PPARγ directly binds to the PPRE in the promoter domain of the *Foxp3* gene (33–35). PPARγ can enhance the transcriptional activity of *Foxp3* by approximately 1.7-fold (49). Furthermore, PPARγ not only directly regulates Foxp3 transcription but also modulates it indirectly (**Figure 5**). Phosphorylated STAT3 (pSTAT3) serves as a transcriptional repressor of *Foxp3*. Experimental evidence from colitis mouse models indicates that activated PPARγ significantly reduces pSTAT3 levels in colonic tissues, thereby abolishing its transcriptional repression of *Foxp3* and facilitating Treg cell differentiation (6, 50). Additionally, PPARγ activation upregulates *CD36/CPT1*-mediated FAO (10), which subsequently promotes the synthesis of UDP-N-acetylglucosamine (UDP-GlcNAc). This cascade enhances the N-glycosylation modification of TβRII/IL-2Rα, activates the TGF-β/Smad3 and IL-2/STAT5 signal cascade, and ultimately increases Foxp3 expression while potentiating Treg cell functional responses (97). Therefore, PPARγ can be utilized to promote Treg cell differentiation. *In vitro* experiments have further validated these regulatory effects. CD3/CD28-stimulated cells cultured with increasing concentrations of the PPARγ agonist PIO for 72 hours demonstrated that PIO significantly elevated the proportion of CD4^+^Foxp3^+^ Treg cells in a dose-dependent fashion (98).

**Figure 5:**
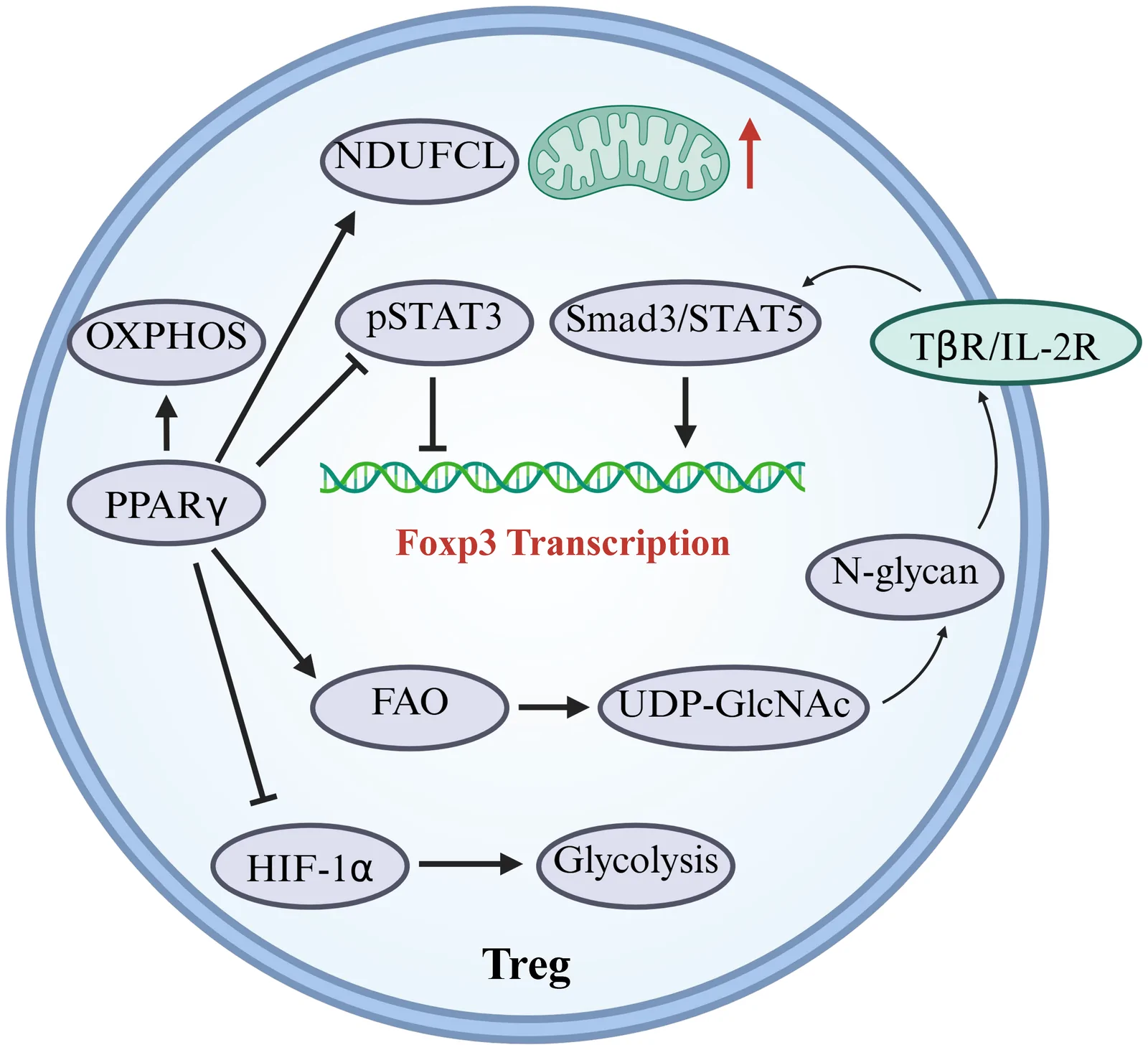
PPARγ in Tregs. This schematic illustrates the PPARγ-mediated metabolic-transcriptional regulatory network in Treg cells. PPARγ boosts OXPHOS and upregulates mitochondrial complex-I subunit *NDUFC1* to sustain mitochondrial fitness. PPARγ also enhances FAO, which generates UDP-GlcNAc to serve as substrate for protein N-glycosylation. N-glycosylation elevates cell-surface abundance of TβRII and IL-2Rα (TβR/IL-2R), amplifying downstream Smad3/STAT5 signaling to promote Foxp3 transcription. PPARγ represses pSTAT3 and relieves its inhibitory effect on Foxp3. Meanwhile, PPARγ inhibits HIF-1α and dampens glycolysis, collectively preserving Treg lineage stability and suppressive function.

Regarding the cytokine microenvironment, PPARγ promotes Treg generation by inducing tolerogenic dendritic cells (DCs) and M2-type macrophages, both of which elaborate TGF-β and IL-10 (32, 33, 36, 37). Mechanistically, PPARγ interacts with NF-κB p65 and STAT3 to suppress pro-inflammatory cytokines, including IL-6, thereby inhibiting Treg-to-Th17 transdifferentiation and maintaining the stability of Foxp3^+^ Treg cells (12, 31). During immunometabolic reprogramming, Treg cells depend on an oxidative metabolic phenotype characterized by activated mitochondrial function, enhanced FAO and OXPHOS, while glycolysis is suppressed (99). Activated PPARγ upregulates several mitochondria-associated genes, including ubiquinone oxidoreductase subunit C1 (*NDUFC1*), and promotes FAO through CPT1 and CD36, thereby sustaining the immunosuppressive function of Treg cells (51, 97). PIO significantly enhances the transcriptional expression of key lipid metabolism-associated genes *CD36* and solute-carrier family 27 member 2 (*Slc27a2*) in cells co-transduced with PPARγ and Foxp3 (52). Butyric acid activates PPARγ and downregulates *HIF-1α*, which inhibits glycolysis, enhances OXPHOS, and promotes the cellular development of naive CD4^+^ Th-cell lineage commitment toward Foxp3^+^ Treg cells (28).

## 7 PPARγ in Th9 cells and Tfh cells

Th9 cells are an essentially Th2-derived subset with high basal PPARγ expression (100), whose expression is further up-regulated during Th9 polarization (38). Endogenously expressed PPARγ fuels activation-triggered aerobic glycolysis through an mTORC1-dependent cascade, selectively elevating IL-9 production while leaving *IL-13* transcription unaltered. By contrast, exogenous synthetic PPARγ agonists upregulate genes governing fatty-acid uptake and engage lipid metabolic circuits to repress IL-9 in a glycolysis-independent manner (38–40). In Th9 cells, PPARγ-governed cystine transport mitigates lipid peroxidation and prevents bioenergetic collapse, thereby sustaining Th9-cell viability and durable effector function. These findings refine the molecular circuitry whereby PPARγ globally underpins the pathogenic potential of Th9 cells (101).

Tfh cells rely on *Bcl-6* as the master transcription factor for their differentiation and produce the key cytokine IL-21 to assist B cells and sustain germinal-center reactions, whereas PPARγ suppresses Tfh-cell differentiation (102). Studies using CD4 T cell-specific PPARγ knockout mice demonstrate for the first time that PPARγ deficiency simultaneously upregulates the expression of Bcl-6 and *IL-21* while reducing *Blimp-1* levels in Th2 cells, and such molecular alterations further drive robust expansion of Tfh cells (41). Enhanced AKT phosphorylation and reduced expression of NF-κB negative regulators, including *IκBα*, *Foxo1* and *Sirt1*, in PPARγ-deficient T cells, which may contribute to augmented Tfh cell generation (18, 41–43). Of note, Park et al. demonstrated that PPARγ-mediated suppression of Tfh responses is sex-dependent: this inhibitory effect is observed exclusively in female mice. In males, single-agent treatment with PPARγ agonists shows no detectable Tfh-suppressing activity, whereas combined administration with 17β-estradiol (ER ligand) restrains Tfh-cell generation. Consistently, female mice under high-estrogen estrus conditions display heightened responsiveness to the PPARγ agonist pioglitazone compared with diestrus females (43).

## 8 PPARγ reshapes Th-cell subset balance

PPARγ shifts the Th1/Th2 immune homeostasis toward a Th2-skewed phenotype (**Figure 6**) (45). In the early stage of Th2 cell activation, glycolysis is required to meet the proliferation needs. In the later stage of differentiation, the FAO pathway is simultaneously upregulated, forming a metabolic pattern of bidirectional balance between glycolysis and FAO (15). In this context, PPARγ mainly governs lipid metabolic rewiring in Th2 cells. PPARγ targets *Fabp4/5*, *CD36* and other genes to maintain Th2 effector activity and tissue residency adaptation (84). The Th2-type cytokines, IL-4/IL-5/IL-13, primarily exert inhibitory effects on Th1 cells (103, 104). Although IFNγ secretion of Th1 is decreased by PPARγ agonists, IFN-γ exerts potent suppression on Th2 cells (105). IFNγ directly inhibits the differentiation of Th17 cells, inducing their apoptosis (106) and promote Treg formation and increases Treg fragility across distinct developmental stages (107, 108). The precise metabolic regulatory pathways mediated by PPARγ in Th1 cells remain to be fully elucidated. Th1 cells mainly rely on glycolysis as the energy production pathway (83). But PPARγ mainly governs the canonical metabolic program that enhances fatty acid uptake, CPT1A-mediated β-oxidation, and intracellular lipid accumulation (109), whereas it barely modulates the T-bet promoter (20).

Treg differentiation is particularly driven by PPARγ, with Treg-derived IL-10 and TGF-β secretion inhibiting Th1/Th2/Th17 differentiation and function in different levels (110–113). The Th17-derived IL-17A can weaken the inhibitory function of Treg cells, key Th1-effector genes and Th2 cytokine production (114–116). PPARγ exerts core metabolic functions by promoting FAO and OXPHOS while repressing glycolysis (28, 29, 97). This metabolic profile aligns with the metabolic demands of Treg cells to facilitate their differentiation (117), yet conflicts with the glycolysis-dependent developmental requirements of Th17 cells to suppress Th17 cell formation, thereby forming the canonical Treg/Th17 metabolic balance axis (**Figure 6**) (118–120).

**Figure 6:**
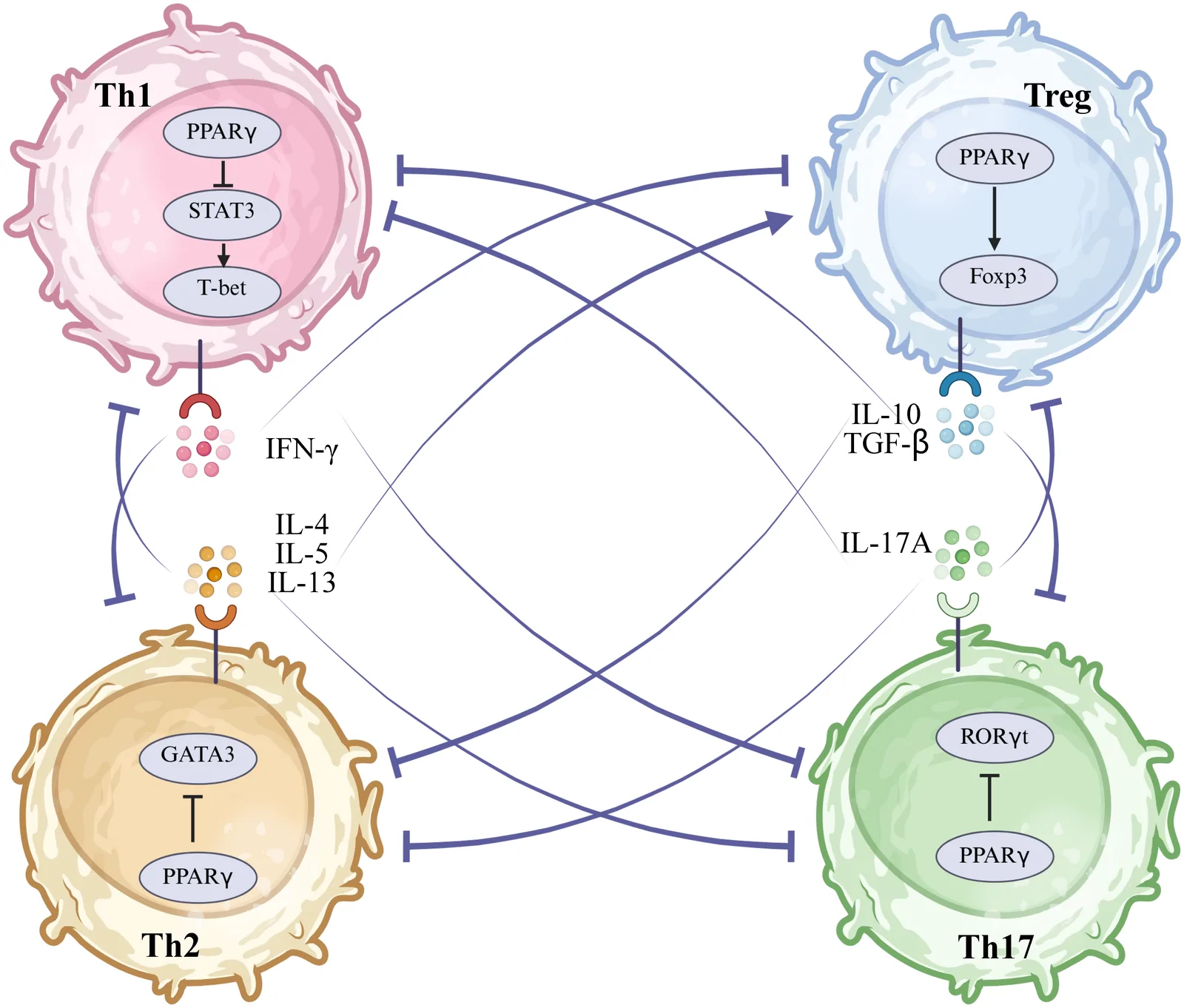
PPARγ regulates the balance of Th-cell subsets. PPARγ inhibits STAT3-T-bet, GATA3, and RORγt signals in Th1, Th2, and Th17 cells, reducing the secretion of pro-inflammatory cytokines such as IFN-γ, IL-4/IL-5/IL-13, and IL-17A; in Treg cells, PPARγ promotes Foxp3 transcription and upregulates inhibitory cytokines such as IL-10 and TGF-β. There is a complex cross-inhibition relationship among CD4^+^ T subsets: the effector cells of Th1/Th2/Th17 mutually antagonize each other, and Treg exert broad-spectrum inhibition on the three types of effector T cells; conversely, the pro-inflammatory factors secreted by the effector T cells can also restrain the function of Treg, jointly maintaining the overall immune homeostasis.

## 9 PPARγ-targeted therapeutic compounds

### 9.1 Rosiglitazone & pioglitazone

Pioglitazone and Rosiglitazone and are TZD PPARγ agonists with similar immunomodulatory profiles but minor mechanistic discrepancies (**Table 3**). In EAE mice, PPARγ overexpression or agonists potently reduce CD4^+^ Th-cell IFN-γ production and Th1 differentiation (127). These two agents can suppress aberrant Tfh cells (137). Early-stage intervention alleviates renal injury in lupus-prone mice, yet they exert only marginal effects on late-stage memory Tfh cells (42). Pioglitazone inhibits Th2-associated GATA-3 transcription factor and effector cytokines IL-4/IL-10 (138). Rosiglitazone has been confirmed to downregulate Th2 signature cytokines IL-4, IL-5 and IL-13 in allergic asthma animal models (121). Both drugs potently and specifically inhibit Th17 (20, 122). They ameliorate various Th17-skewed inflammatory conditions such as obesity-linked psoriasis (139), metabolic vascular inflammation, systemic lupus erythematosus (SLE) (140) with variable efficacy in preclinical researches. Rosiglitazone acts as a critical positive regulator of Treg homeostasis. It significantly upregulates Foxp3 expression, expands the pool of functional Tregs in adipose tissue and peripheral circulation, elevates IL-10 secretion, and potentiates the immunosuppressive capacity of Tregs (97).

Pioglitazone has phase III clinical evidence, approved for type 2 diabetes (T2DM) (128), with phase II trials targeting metabolic dysfunction-associated steatotic liver disease (MASLD/MASH) (129). Beyond glycemic control (123), phase II trials explored the potential of rosiglitazone in NASH and mild-to-moderate ulcerative colitis (UC) (124, 125), yet neither indication proceeded to phase III or obtained regulatory approval.

Due to historical cardiovascular safety concerns, rosiglitazone lacks large-scale modern metabolic trials. Repurposing-oriented phase I/II trials (NCT04114136) are investigating rosiglitazone combined with anti-PD-1 agents for advanced solid tumors including hepatocellular carcinoma, non-small-cell lung cancer and melanoma.

### 9.2 Lobeglitazone

Lobeglitazone is a dual PPARα/γ agonist of TZDs (**Table 3**). It suppresses Th2 responses (reduced IL-5, IL-13 and serum IgE) and alleviates airway inflammation and mucus hypersecretion in Ovalbumin-challenged (OVA) asthmatic mice, via inhibiting NF-κB phosphorylation and AP-1 expression (131). It is currently marketed in South Korea for type 2 diabetes mellitus (141).

Phase I-III clinical trials demonstrated its comparable glycemic efficacy versus rosiglitazone and pioglitazone, with markedly lower incidence of edema, weight gain and fracture risk (132, 133). Phase II pilot study suggested beneficial effects on NAFLD (134).

### 9.3 GW9662

GW9662 acts as an irreversible covalent PPARγ antagonist (**Table 3**) (142). In co-cultures of naive CD4^+^ T cells treated with PPARγ agonists, GW9662 reversed agonist-mediated inhibition of Th17 cell differentiation (increased IL-17A) (135) and abolished agonist-driven Treg induction (decreased Foxp3) (97).The compound itself has pharmacokinetic and drug-likeness deficiencies, and thus has not been developed into a clinical drug.

### 9.4 T0070907

T0070907 is a high-affinity irreversible PPARγ antagonist (**Table 3**) (143). In *in vitro* polarization assays of Th17/Treg cells using murine splenic T cells, it acts similarly to GW9662 to block PPARγ agonist-mediated Th17 reduction and Treg elevation, thereby increasing the proportion of Th17 cells (136). This substance is merely a preclinical research tool compound and has not been advanced for human trials.

**Table 3:** Summary of PPARγ-targeted therapeutic compounds.

Compounds	Effects on Th-cell subsets	Preclinical studies	Clinical trials	Ref
Rosiglitazone	-Th1 -Th2 -Th17 +Treg -Tfh	Allergic asthma DSS colitis HDM asthma SLE (MRL/lpr)	T2DM (Phase III) NASH (Phase II) UC (Phase II)	(65, 121–126)
Pioglitazone	-Th1 -Th2 -Th17 +Treg -Tfh	ApoE^-^/^-^ atherosclerosis EAE (Multiple Sclerosis)	T2DM (Phase III) MASLD/MASH (Phase II)	(127–130)
Lobeglitazone	-Th2	OVA asthma	T2DM (Phase III) NAFLD (Phase II)	(131–134)
GW9662	+Th17 -Treg	DSS colitis Neutrophilic asthma	NA	(97, 135)
T0070907	+Th17 -Treg	Mouse splenic T cell	NA	(136)

This table summarizes the research findings of 3 PPARγ full agonists (Rosiglitazone, Pioglitazone, Lobeglitazone) and 2 PPARγ antagonists (GW9662, T0070907) in the regulation of Th-cell subgroups, preclinical disease models, and clinical trial stages.

## 10 Limitations and future directions

Although the modulatory capacity of PPARγ in Th-cell subsets has been elucidated, several limitations persist. Current research in this field is still dominated by phenomenological description and preliminary exploration of basic mechanisms, lacking a complete, accurate, and practicable regulatory theoretical system. There remain prominent deficiencies in the in-depth mechanistic interpretation and clinical translational application of relevant research. First, the context-dependent bidirectional regulatory mechanism has not yet been clarified. Existing studies hold conflicting views on the regulatory effect of PPARγ on Th2 cells. Some investigations have demonstrated that PPARγ facilitates Th2 cell differentiation via the transrepression pathway, whereas other studies have verified that PPARγ binds to NFAT, thereby inhibiting the expression of Th2 signature cytokines including IL-4, IL-5, and IL-13 (63). PPARγ also demonstrates significant gender specificity in regulating effector Th-cell differentiation (144). Secondly, the coverage of Th-cell subsets and signaling pathways in current research remains insufficient. Existing studies are overwhelmingly restricted to the four canonical CD4^+^ helper T subsets, namely Th1, Th2, Th17 and Tregs, while investigations into non-classical subsets including Tfh, Th9 and Th22 are fragmented and lack systematic characterization. Findings from basic research are hard to translate into clinical practice. Species discrepancies, insufficient clinical evidence and drug toxicity severely restrict the translational value of PPARγ-targeted immunotherapy. First, a major translational gap exists between mouse models and the human immune system. Most current regulatory mechanism findings are obtained from mouse Th-cells *in vitro* assays and knockout models, with limited validations in primary and tissue-infiltrating Th-cells from patients with autoimmune and allergic diseases (145). Conventional PPARγ agonists and antagonists show poor *in vivo* performance. Th-cell-selective modulators without systemic metabolic side effects are still lacking, severely restricting the safety and practical utility of clinical regimens (62, 146). Secondly, preclinical studies predominantly test PPARγ modulators in mouse autoimmune/inflammatory disease models, whereas clinically approved indications for these PPARγ agonists are limited to type 2 diabetes. Human trials seldom adopt Th-cell subset balance as formal endpoints; hence, it remains unknown whether mouse-observed Th-cell rebalancing occurs in human autoimmune patients. Future clinical studies of PPARγ-targeted therapies for autoimmunity should include Th-cell-focused immunophenotyping (circulating/tissue-infiltrating Th1/Th2/Th17/Treg frequencies, signature cytokines, lineage-specific transcription-factor expression) as trial readouts. These Th-cell-associated biomarkers can validate the translational fidelity of preclinical animal models and clarify whether PPARγ-driven helper-T-cell remodeling yields clinical benefits in human autoimmune diseases.

Based on current mechanistic evidence, future research should focus on standardized mechanistic assays, PPARγ-targeted nano-delivery systems, and corresponding cell engineering therapies. First, Standardized procedures for simultaneous tissue collection across multiple tissues and unified flow cytometric gating criteria for all tissues should be established in research to eliminate detection biases caused by tissue context dependency (82). Second, given the systemic metabolic side effects of thiazolidinediones, next generation nanomedicine platforms, including PLGA-encapsulated PPARγ agonists, lipid nanoparticle (LNP) or exosome-based targeted delivery functionalized with anti-CD4 or Treg homing peptides (e.g., CCR7/α4β7 ligands), should be developed to achieve Treg-selective immunometabolic reprogramming while sparing adipocyte PPARγ activation (78). Third, future studies should explore T cell intrinsic PPARγ engineering: PPARγ overexpression or knockout in human primary T cells or CAR-T constructs could dissect whether PPARγ-driven metabolic change enhances CAR-T stem-like memory phenotype and persistence, or conversely whether PPARγ inhibition in Th17/Tfh subsets reduces exhaustion in solid-tumor CAR-T models (147).

## 11 Conclusion

This paper provides a systematic review of the regulatory mechanisms by which PPARγ influences various Th-cell subsets. It emphasizes the core mechanisms involved in regulating key transcription factors, metabolic reprogramming, and the cytokine microenvironment. Future research should prioritize elucidating context-dependent mechanisms, developing Th-cell-specific PPARγ modulators, investigating interactions with signaling pathways, and broadening studies to include other Th-cell subsets. These advancements will facilitate the restoration of Th-cell homeostasis, enhance the specificity and safety of targeted therapies, and offer new strategies for managing refractory diseases. In addition, the cell therapy methods targeting and regulating PPARγ remain to be further explored. Combining the PPARγ target, gene editing technology, and CAR-T cell therapy and other cutting-edge approaches may have a high potential for application. This field holds significant academic and clinical value and will contribute to the prevention and therapeutic intervention of immune-related disorders.
